# Comparison of pH Data Measured with a pH Sensor Array Using Different Data Fusion Methods

**DOI:** 10.3390/s120912098

**Published:** 2012-09-04

**Authors:** Yi-Hung Liao, Jung-Chuan Chou

**Affiliations:** 1 Department of Information Management, TransWorld University, 1221 Zhennan Rd., Yunlin 64063, Taiwan; E-Mail: liaoih@twu.edu.tw; 2 Graduate School of Electronic and Optoelectronic Engineering, National Yunlin University of Science and Technology, 123 University Rd., Yunlin 64002, Taiwan

**Keywords:** data fusion, sensor array, electrochemical, ruthenium dioxide

## Abstract

This paper introduces different data fusion methods which are used for an electrochemical measurement using a sensor array. In this study, we used ruthenium dioxide sensing membrane pH electrodes to form a sensor array. The sensor array was used for detecting the pH values of grape wine, generic cola drink and bottled base water. The measured pH data were used for data fusion methods to increase the reliability of the measured results, and we also compared the fusion results with other different data fusion methods.

## 1. Introduction

The investigation of data fusion has developed since the 1980s. The United States Department of Defense (DoD) first used data fusion for a military detection and management system [1]. In recent years, data fusion has been applied to various application fields, such as robotics, image processing and non-military purposes, and is also used in traffic management and smart transport systems. Sharma and Raju [2] have described some characteristics of data fusion as follows: it raises information reliability, reduces uncertainty, improves detection effects, increases practicability, *etc.*, as in weighted average methods [3–6], fuzzy fusion and neural network fusion [7]. This paper introduces some data fusion methods in later sections, consisting of average data fusion, self-adaptive data fusion [3], fuzzy set data fusion [8] and coefficient of variance data fusion [9]. Li *et al.* [10] stated that sensor networks were an integration of sensor techniques, nested computation techniques, distributed computation techniques and wireless communication techniques. They can be used for testing, sensing, collecting and processing information of monitored objects and transferring the processed information to users. Sensor networks represent a new research area of computer science and technology and have wide application in the future. Both academia and industries are very interesting in them. The concepts and characteristics of sensor networks and the data in the networks were introduced, and the issues of the sensor networks and the data management of sensor networks were discussed. The advances of research on sensor networks and the data management of sensor networks were also presented. Wang *et al.* [11] proposed a new mobile-agent-based adaptive data fusion (ADF) algorithm to determine the minimum number of measurements each node required for a perfectly joint reconstruction of multiple signal ensembles. They theoretically showed that ADF provided the optimal strategy with the minimum total number of measurements possible and hence reduced communication cost and network load.

Xia *et al.* [12] introduced a novel approach called the linearly constrained least squares (LCLS) method for statistical data fusion. The LCLS method uses only the constrained minimum sample variance of fused information, and the proposed fusion method can tackle the unknown covariance problem. Wei [13] introduced that multi-sensor data fusion technology was one of the main techniques of the modern C31 system, and the C31 system performance played a decisive role. The paper used Visual C++ and MATLAB languages to jointly design and construct a universal visualization multi-sensor data fusion simulation platform, which provided researchers with a variety of fusion algorithm simulations and quantitative assessment of the simulation environment, as well as carrying out teaching and scientific research to provide support. Recently, Zakaria *et al.* [14] reported an improved classification of the herb *Orthosiphon stamineus* using a data fusion technique. Low level fusion was performed by combining the information provided by different sensors in different modalities. Principal component analysis (PCA) and linear discriminant analysis (LDA) were chosen to perform the low level fusion.

Utilization of data sources measured with a sensor array in pH sensing studies has gained popularity with recent technological advances. Data fusion was used to provide a better solution than could otherwise be achieved from the use of single sensor data alone. Data fusion was used to produce an improved model or estimate of a sensing system from a set of independent data sources. In this study, we investigated the feasibility of using the data fusion method for a pH sensor array and used the measured pH data to apply these data fusion methods. This research investigated the comparison of data measured by the electrochemical pH sensor array with different data fusion methods. The primary objective of this study was to select an appropriate data fusion method for electrochemical measurement applications, regardless of whether the pH sensor array contained a failed pH sensor.

## 2. Experimental

**Figure 1.**
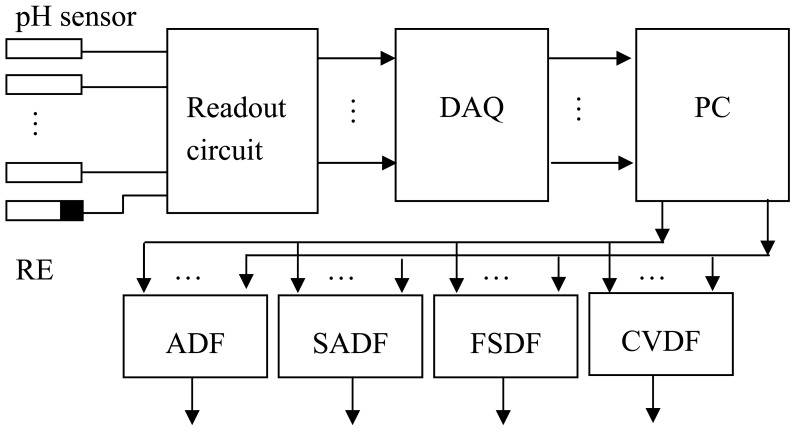
Experimental structure includes a sensor array with eight pH sensors, a reference electrode, readout circuit and uses a data acquisition card connected to a personal computer, and the measured data used for data fusion with different data fusion methods.

In this paper, the sensor array for pH measurement was based on the ruthenium dioxide (RuO_2_) pH electrode. The RuO_2_ thin film was deposited onto a silicon substrate using a sputtering system. In the experimental process, the sensor array and the Ag/AgCl reference electrode (RE) were immersed in commercial drinks (grape wine, generic cola drink and bottled base water) to obtain the pH readings by using a voltage-time measurement system interfaced with the program LabVIEW. The experiment uses a sensor array (eight pH electrodes) and repeats measurements fifteen times [15]. Figure 1 show a sensor array with eight pH sensors, a reference electrode, readout circuit, and the data acquisition card connected to a personal computer. The measured data were used for data fusion with different data fusion methods which are average data fusion (ADF), self-adaptive data fusion (SADF), fuzzy set data fusion (FSDA), and coefficient of variance data fusion (CVDF). The readout circuit consists of eight instrument amplifiers (IAs) and low pass filters (LPFs). The DAQ card is a product of National Instrument (NI) with universal series bus (USB) interface.

## 3. Data Fusion Methods

### 3.1. Average Data Fusion (ADF)

The pH measured data were obtained from a pH sensor array with eight ruthenium dioxide pH electrodes. For example, each sensor was measured n times during a measurement period. The pH measured data were used as a mathematical or statistical method to obtain the average value (mean) for the data measured n times from each pH sensor. Let the n times measured data of the *i*^th^ sensor and the mean (μ) of n times measured data of the *i*^th^ sensor be as follows [15]:

(1) $$x_{i}(k),k=1,2,\cdots ,n$$

(2) $$\mu_{i}=\frac{1}{n}\sum_{k=1}^{n}x_{i,k}$$

where *i* is the number of sensors, *k* is the number of data measurements for each pH sensor.

In this study, the average data fusion of sensor array with eight pH electrodes have the same weighted coefficients (*w*_1_ = *w*_2_ =⋯= *w*_8_). The sum of weighted coefficients is equal to 1 and the final average data fusion of sensor array is shown as follows [15]:

(3) $$\sum_{i=1}^{8}w_{i}=1$$

(4) $$\mu_{\text{ADF}}=\frac{1}{8}\sum_{i=1}^{8}\mu_{i}$$

### 3.2. Self-Adaptive Data Fusion (SADF)

The current work obtained pH measured values from eight ruthenium dioxide pH sensors. Each sensor was measured n times during a measurement period. The pH measured data can be pre-processed using a mathematical or statistical method to obtain the mean (*ȳ_i_*) and variance $\left(\sigma_{i}^{2}\right)$ for the n times measured data from each pH sensor. The mean and variance equations are expressed in general form as follows [3]:

(5) $${\bar{y}}_{i}=\frac{1}{n}\sum_{j=1}^{n}y_{ij}$$

(6) $$\sigma_{i}^{2}=\frac{1}{n-1}\sum_{j=1}^{n}{\left(y_{ij}-{\bar{y}}_{i}\right)}^{2}$$

where *i* is the number of sensors, *j* is the number of data measurements for each pH sensor, *y_ij_* is the *j*^th^ data from the *i*^th^ sensor, *ȳ_i_* and $\sigma_{i}^{2}$ are the mean and variance from the *i*^th^ sensor, respectively.

Here, we utilize the sensor array based on the minimum mean variance to proceed with measurement data fusion. First, we assume that all data from each sensor have the same mean and exclusion independent each other. We evaluated the weighted coefficients *w*_i_ (*w*_1_, *w*_2_, … *w*_n_) for each pH sensor and the sum of weighted factors for each pH sensor is equal to unity. The estimated data fusion value *μ_y_* can then be described as follows [3]:

(7) $$\sum_{i=1}^{n}w_{i}=1$$

(8) $$\mu_{y}=\sum_{i=1}^{n}w_{i}y_{i}$$

where *w*_i_ is the weighted coefficient of the *i*^th^ sensor, *y_i_* is the measured data of the *i*^th^ sensor, *μ_y_* is the final value after data fusion. After data fusion, the equation for the total mean variance is as follows [3]:

(9) $$\begin{array}{ll} \sigma^{2} & =\;E[{\left(Y-\mu_{y}\right)}^{2}]=E[{\left(Y-\sum_{i=1}^{n}w_{i}y_{i}\right)}^{2}]\\ & =\;E[{\left(Y\sum_{i=1}^{n}w_{i}-\sum_{i=1}^{n}w_{i}y_{i}\right)}^{2}]=E[{\left(\sum_{i=1}^{n}w_{i}Y-\sum w_{i}y_{i}\right)}^{2}]\\ & =\;E[{\left(\sum_{i=1}^{n}w_{i}(Y-y_{i})\right)}^{2}]=\sum_{i=1}^{n}w_{i}^{2}\sigma_{i}^{2} \end{array}$$

From Equation (6), this study can obtain the total of mean variance *σ*^2^ which is related to each weighted coefficient in the multi-dimension second order function. According to the multi-dimension function theory, we can obtain the *f* function that consists of *λ* and *w*_i_ variables in the equation as follows [3]:

(10) $$f(w_{1},w_{2},\cdots ,w_{n},\lambda )=\sum_{i=1}^{n}w_{i}^{2}\sigma_{i}^{2}+\lambda (\sum_{i=1}^{n}w_{i}-1)$$

The Lagrange multiplier method is used to evaluate the solution of Equation (9). Let the *f* function proceeds partial deviation of λ and *w*_i_, respectively. The equations are obtained as follows [3]:

(11) $$\frac{\partial f}{\partial w_{i}}=2w_{i}\sigma_{i}^{2}-\lambda =0(i=1,2,\cdots ,\text{n})$$

(12) $$\frac{\partial f}{\partial \lambda }=1-\sum_{i=1}^{n}w_{i}=0$$

The solutions of the Equations (11) and (12) are evaluated and the expressed equation for *w*_i_ is obtained as follows [3]:

(13) $$w_{i}=\frac{1}{\sigma_{i}^{2}\left(\overset{n}{\underset{k=1}{\text{∑}}}\sigma_{k}^{-2}\right)}(i=1,2,\cdots ,\text{n})$$

### 3.3. Fuzzy Set Data Fusion (FSDA)

There are n sensors in the measurement system and the sensors are used to determine the analyte, respectively. The measured values of the *i^th^* sensor in the *k* time are shown as follows [8]:

(14) $$x_{i}(k),i=1,2,\cdots ,n$$

The measured values of each sensor acted as a fuzzy set. According to the fuzzy mathematic theory, we can closely measure between two fuzzy sets.

Definition 1: the approach degree measured values of the *i* sensor and *j* sensor at *k* time is shown as follows [8]:

(15) $$\sigma_{ij}(k)=\min\{x_{i}(k),x_{j}(k)\}/\max\{x_{i}(k),x_{j}(k)\}$$

Definition 2: the approach degree matrix between each sensor at *k* time is shown as follows [8]:

(16) $$\sum (k)=\left[\begin{array}{cccc} 1 & \sigma_{12}(k) & \cdots & \sigma_{1n}(k)\\ \sigma_{21}(k) & 1 & \cdots & \sigma_{2n}(k)\\ \vdots & \vdots & \ddots & \vdots \\ \sigma_{n1}(k) & \sigma_{n2}(k) & \cdots & 1 \end{array}\right]$$

Definition 3: the consistence measurement of the measured value between the *i^th^* sensor and other sensors in the time of *k* is shown as follows [8]:

(17) $$r_{i}(k)=\sum_{j=1}^{n}\sigma_{ij}(k)/n$$

Considering measurement region dependability and defining the mean and variance of the *i^th^* sensor are shown as follows [8]:

(18) $${\bar{r}}_{i}(k)=\sum_{i=1}^{k}r_{i}(k)/k$$

(19) $$\sigma_{i}^{2}(k)=\sum_{i=1}^{k}{\left[r_{i}(t)-{\bar{r}}_{i}(t)\right]}^{2}/k$$

Definition 4: the measurement of consistence dependability of the *i^th^* sensor in the time of *k* is shown as follows [8]:

(20) $$w_{i}(k)={\bar{r}}_{i}(k)/\sigma_{i}^{2}(k)$$

Regularity equal to one then we have the following form [8]:

(21) $$W_{i}(k)=w_{i}(k)/\sum_{j=1}^{n}w_{j}(k)$$

Utilizing the measurement of consistence dependability for data fusion, to obtain the measured value of data fusion of all sensors in the *k* time is presented as follows [8]:

(22) $$x_{f}(k)=\sum_{i=1}^{n}W_{i}(k)x_{i}(k)$$

### 3.4. Coefficient of Variance Data Fusion (CVDF)

The coefficient of variance (CV), also named discrete coefficient, is used for different measurement data. The CV is the ratio of the standard deviation and mean value. The *CV_i_* is presented as the coefficient of variance of measured data *X_i_*, and the calculation of the *CV_i_* is described as follows [9]:

(23) $$CV_{i}=\sigma_{i}/\mu_{i}$$

To utilize the coefficient of variance for the data fusion of sensor array, the data process and equation are shown as follows [9]:

1. From Equation (23), calculate the coefficient of variance with measured data of sensor array (*CV*_1_, *CV*_2_, ⋯, *CV*_n_).
2. Calculate the reciprocal of the coefficient of variance with measured data of sensor array ( $CV_{1}^{-1},CV_{2}^{-1},\cdots ,CV_{n}^{-1}$).
3. Let the reciprocal of the coefficient of variance, to obtain the weighting fusion of sensor array.
  (24) $$w_{i}=CV_{i}^{-1}/\sum_{j=1}^{n}CV_{j}^{-1},i=1,\cdots ,n$$
4. The result of fusion is described as follows [9]:
  (25) $$x_{f}=\sum_{i=1}^{n}w_{i}\mu_{i}$$

## 4. Results and Discussion

This study can obtain the pH measurement data of grape wine, generic cola drink and bottled base water from the ruthenium dioxide sensor array as shown in Table 1. We utilized the pH measured data of drinks in Table 1 to obtain fusion results with different data fusion methods. We also compared the fusion results with average data fusion, self-adaptive data fusion, fuzzy set data fusion and coefficient of variance data fusion *etc.* The pH sensor array was measured one time and obtained eight pH data. In this research, we provided an appropriate data fusion method for electrochemical measurement applications. This study is associated the various data fusion methods and pH sensor array to investigate the reliability of measured results of sensor array and without removing the measured data of the failed pH sensor among sensor array.

### 4.1. Average Data Fusion (ADF)

The current study has obtained average data fusion (ADF) from each sensor with the same weighted coefficients (*w*_1_ = *w*_2_ =⋯= *w*_8_). We used Equation (4) to derive the average data fusion with the pH measured data. The weighted coefficient of eight sensor array is 0.125. The data fusion results of grape wine, generic cola drink and bottled base water with average data fusion are 4.04, 5.11 and 7.62, respectively, and are shown in Table 2.

### 4.2. Self-Adaptive Data Fusion (SADF)

The measured pH data of the RuO_2_ sensor array were used with self-adaptive data fusion (SADF). We used the Equation (13) to obtain the weighted coefficients (*w_i_*) of sensor array. The weighted coefficients and the fusion results with weighted coefficients of SADF are shown in Table 3. The fusion results of grape wine, generic cola drink and bottled base water by using self-adaptive data fusion are 3.58, 4.67 and 7.44, respectively.

### 4.3. Fuzzy Set Data Fusion (FSDF)

We used Equation (21) to obtain the weighted coefficients (*w_i_*) of the sensor array with fuzzy set data fusion (FSDF). The fusion results and weighted coefficients (*w_i_*) of every sensor are shown in Table 4. The fusion results of grape wine, generic cola drink and bottled base water with fuzzy set data fusion are 3.56, 4.68 and 7.30, respectively.

### 4.4. Coefficient of Variance Data Fusion (CVDF)

We used Equation (24) to obtain the weighted coefficients (w_i_) of the sensor array with coefficient of variance data fusion (CVDF). Table 5 shows the fusion results of the coefficient of variance data fusion with weighted coefficients (w_i_) of every sensor. The measured pH data of grape wine after coefficient of variance data fusion is 3.62. The measured pH data of generic cola drink after coefficient of variance data fusion is 4.79 and the measured pH data of bottled base water after coefficient of variance data fusion is 7.46.

### 4.5. Summary Results

The weighted coefficients of various data fusion methods are obtained from the measured pH data and used these measured pH data to compute the mean (μ), standard deviation (σ) and variance (σ^2^) with mathematic statistic functions. In this study, we investigated the various data fusion methods and applied for the measured pH values of an electrochemical pH sensor array. The fusion technology is only to use the measured data and added mathematic statistic formula to derive the solution. Table 6 shows the summary of pre-calculation, weighted coefficient and computational complexity with different data fusion methods. The mean (μ) value of measured data was obtained for the H sensor array in the average data fusion in advance. The average data fusion has the same weighted coefficient and the complexity of calculation is easy. The self-adaptive data fusion need to obtain the mean (μ) and variance (σ^2^) beforehand, the weighted coefficients of sensor array were obtained from the variance of each pH sensor. The approach degree (σ_ij_) and consistent (r_i_) were used to calculate the weighted coefficients of the fuzzy set data fusion. The coefficient of variance data fusion used the mean (μ) and standard deviation (σ) to derive the weighted coefficients for pH sensor array. According to the computational process of these data fusion methods, in which the fuzzy set data fusion uses the variance and matrix operations and is more difficult than the others. The computational complexity of self-adaptive and coefficient of variance data fusions are moderate with mean, standard deviation and variance statistic operation. The average data fusion has easy computation with arithmetic average to get the weighted coefficients.

In this study, we have performed a series of trials for commercial drinks using different data fusion methods with a RuO_2_ pH sensor array. This section summarizes the fusion results in this experiment. We used the measured pH data of Table 1 for different data fusion methods to perform the data fusion. Tables 2–5 present the experiment results. Table 2 shows the final results of the pH measurement of grape wine, generic cola drink and bottled base water with a single sensor. The no. 6 sensor failed and its measured value is very different from other sensors. The fusion results with different data fusion methods are shown in Table 7 and compared with the measurement results of a commercial pH meter. From Table 7, we can conclude that the fusion result of average data fusion is more different from the commercial pH meter than the other data fusion methods. This phenomenon is due to the fact the 6th sensor of the pH sensor array failed; its measured data was incorrect and had the same weight coefficient as the others. As to the other data fusion methods, the 6th sensor has a smaller weighted coefficient, therefore the fusion results were fairly close to the measured value of the commercial pH meter. According to the experimental results, the conclusion was that the fusion results with self-adaptive, fuzzy set and coefficient of variance methods were superior to a single failed pH sensor and the average data fusion.

## 5. Conclusions

This study used ruthenium dioxide pH electrodes to form a sensor array and obtained a set of measured pH data with a voltage-time measurement system. The sensor array was applied to measure the pH of commercial drinks. The measured pH data were used for different data fusion methods. We also compared the fusion results with different data fusion methods and investigated the complexity of each one. The data fusion results were obviously superior to a single failed sensor and the average data fusion.

## Figures and Tables

**Table 1.** The pH measured data of grape wine, generic cola drink and bottled base water drinks with the sensor array used for different data fusion methods.

Times		1	2	3	4	5	6	7	8	9	10	11	12	13	14	15
Sensors																
Grape wine	1	3.85	3.82	3.79	3.68	3.98	3.50	3.86	3.63	3.53	3.89	3.74	3.46	3.67	3.75	3.66
	2	3.75	3.64	3.58	3.82	3.69	3.76	3.66	3.58	3.71	3.64	3.81	3.61	3.71	3.67	3.71
	3	3.72	3.70	3.73	3.73	3.77	3.64	3.67	3.52	3.78	3.73	3.70	3.73	3.73	3.75	3.74
	4	3.32	3.33	3.27	3.38	3.58	3.36	3.29	3.29	3.41	3.35	3.46	3.39	3.36	3.44	3.37
	5	3.41	3.48	3.57	3.54	3.75	3.59	3.57	3.54	3.62	3.52	3.44	3.53	3.59	3.57	3.59
	(6) *	4.80	4.83	5.83	5.67	8.72	10.21	10.12	10.00	9.80	9.02	8.24	7.52	6.36	5.16	4.37
	7	3.56	3.59	3.45	3.31	3.30	3.27	3.10	3.15	3.29	3.59	3.76	3.66	4.01	3.99	3.46
	8	3.57	3.59	3.56	3.49	3.51	3.48	3.39	3.40	3.38	3.39	3.40	3.38	3.37	3.36	3.36
Generic cola drink	1	4.26	4.37	4.58	4.73	4.63	4.46	4.67	4.60	4.70	4.63	4.21	4.02	4.23	4.71	4.70
	2	4.20	4.35	4.43	4.71	4.82	4.80	4.81	4.83	4.91	5.02	4.96	4.96	4.90	4.87	4.74
	3	4.15	4.52	4.63	4.64	4.88	4.86	4.80	4.76	4.87	4.73	4.75	4.79	4.85	4.88	4.76
	4	3.98	4.34	4.60	4.58	4.70	4.77	4.89	4.76	4.74	4.79	4.76	4.80	4.79	4.72	4.76
	5	4.08	4.35	4.54	4.63	4.65	4.64	4.67	4.71	4.71	4.74	4.73	4.74	4.76	4.73	4.72
	(6) *	5.83	8.04	9.01	7.22	8.39	9.12	9.52	9.46	9.36	9.35	9.22	9.01	8.96	5.35	6.59
	7	3.98	4.32	4.40	4.43	4.88	4.72	4.74	4.61	4.72	4.79	4.75	4.79	4.76	4.63	4.64
	8	4.82	4.16	4.26	4.58	4.55	4.82	4.85	4.79	4.79	4.80	4.82	4.76	4.79	4.72	4.81
Bottled base water	1	7.80	7.72	7.69	7.90	7.97	7.56	7.86	7.81	7.71	7.67	7.81	7.77	7.91	7.63	7.62
	2	7.67	6.99	7.53	7.13	7.09	7.09	7.11	7.36	7.41	7.28	7.36	7.46	7.15	7.23	7.19
	3	6.91	7.39	7.58	7.11	7.13	7.28	7.31	7.27	7.23	7.43	7.33	7.22	7.26	7.27	7.37
	4	7.73	7.05	7.81	7.39	6.91	6.98	7.14	7.25	6.98	7.14	7.00	7.02	7.13	7.14	7.09
	5	7.77	7.25	7.30	7.53	7.04	6.99	7.13	7.38	7.34	7.47	7.66	7.66	7.52	7.44	7.23
	(6) *	9.58	8.39	10.32	11.54	10.18	9.38	8.76	9.11	8.58	8.30	9.57	10.26	11.55	9.66	8.33
	7	7.15	6.81	7.05	7.16	7.00	7.49	7.15	7.08	7.59	7.31	7.22	7.36	7.49	7.02	6.99
	8	7.57	7.10	7.32	7.63	7.45	7.22	7.36	7.58	7.18	7.06	7.16	7.23	7.33	7.15	7.65

* Sensor No. (6) failed.

**Table 2.** The pH measured data of grape wine, generic cola drink and bottled base water drinks with sensor array and used average data fusion (ADF) to obtain fusion results.

**i**	**1**	**2**	**3**	**4**	**5**	**6**	**7**	**8**	**ADF**
Grape wine	3.72	3.69	3.71	3.37	3.55	**7.38**	3.50	3.44	4.04
Generic cola drink	4.50	4.75	4.73	4.67	4.63	**8.29**	4.61	4.69	5.11
Bottled base water	7.76	7.27	7.27	7.18	7.38	**9.57**	7.19	7.33	7.62

**Table 3.** The weighted coefficients of self-adaptive data fusion (SADF) for every sensor and used the pH measured data of grape wine, generic cola drink and bottled base water drinks to obtain fusion results.

**Sensor (i)**	**Grape wine (w_i_)**	**Generic cola drink (w_i_)**	**Bottled base water (w_i_)**
1	0.045372	0.127247	0.324111
2	0.187159	0.111269	0.123980
3	0.272164	0.179767	0.190449
4	0.164172	0.123371	0.063876
5	0.162918	0.196128	0.085280
6	0.000218	0.003356	0.004028
7	0.013709	0.114756	0.095197
8	0.154288	0.144106	0.113079

SADF	3.58	4.67	7.44

**Table 4.** The weighted coefficients of fuzzy set data fusion (FSDF) for every sensor and used the pH measured data of grape wine, generic cola drink and bottled base water drinks to obtain fusion results.

**Sensor (i)**	**Grape wine (w_i_)**	**Generic cola drink (w_i_)**	**Bottled base water (w_i_)**
1	0.105599	0.024853	0.027837
2	0.130574	0.129387	0.122701
3	0.146149	0.190182	0.100886
4	0.232531	0.104639	0.172091
5	0.188192	0.168873	0.165584
6	0.002562	0.003567	0.004394
7	0.059306	0.286215	0.172905
8	0.135085	0.092284	0.233602

FSDF	3.56	4.68	7.30

**Table 5.** The weighted coefficients of coefficient of variance data fusion (CVDF) for every sensor and used the pH measured data of grape wine, generic cola drink and bottled base water drinks to obtain fusion results.

**Sensor (i)**	**Grape wine (w_i_)**	**Generic cola drink (w_i_)**	**Bottled base water (w_i_)**
1	0.088093	0.126295	0.227577
2	0.177495	0.124831	0.131845
3	0.215109	0.157725	0.163473
4	0.151923	0.129017	0.093518
5	0.159425	0.161257	0.111017
6	0.012109	0.037826	0.031278
7	0.045561	0.122942	0.114288
8	0.150284	0.140107	0.127005

CVDF	3.62	4.79	7.46

**Table 6.** The summary of pre-calculation, weighted coefficient and computational complexity with different data fusion methods *.

**Methods**	**Pre-Calculation**	**Weighted Coefficient (w_i_)**	**Computational Complexity**
ADF	μ	1/n	easy
SADF	μ,σ^2^	$\frac{1}{\sigma_{i}^{2}\left(\overset{n}{\underset{k=1}{\text{∑}}}\sigma_{k}^{-2}\right)}$	moderate
FSDF	*σ_ij_, r_i_, r̄_i_*, $\sigma_{i}^{2}$	$\begin{array}{c} w_{i}(k)={\bar{r}}_{i}(k)/\sigma_{i}^{2}(k)\\ w_{i}(k)/\overset{n}{\underset{j=1}{\text{∑}}}w_{j}(k) \end{array}$	difficult
CVDF	μ,σ, CV = σ/μ	$CV_{i}^{-1}/\overset{n}{\underset{j=1}{\text{∑}}}CV_{j}^{-1}$	moderate

* μ: mean; σ: standard deviation; σ^2^: variance; σ_ij_: approach degree; *r_i_*: consistent; *r̄_i_*: mean of consistent; n: sensor amount.

**Table 7.** Comparison of the fusion results with pH measured data fusion of grape wine, generic cola drink and bottled base water drinks with different data fusion methods.

**Fusion Method**	**Grape Wine**	**Generic Cola Drink**	**Bottled Base Water**
Average data fusion (ADF)	4.04	5.11	7.62
Self-adaptive data fusion (SADF)	3.58	4.67	7.44
Fuzzy set data fusion (FSDF)	3.56	4.68	7.30
Coefficient of variance data fusion (CVDF)	3.62	4.79	7.46
Commercial pH meter	3.61	4.24	7.32
